# Advancement of a RGBW-LED pen for diaphanoscopic illumination with adjustable color and intensity with tests on ex-vivo porcine eyes in terms of retinal risk and correlated color temperature

**DOI:** 10.1007/s13534-023-00317-4

**Published:** 2023-09-15

**Authors:** Nicole Fehler, David Schneider, Martin Hessling

**Affiliations:** Institute of Medical Engineering and Mechatronics, Ulm University of Applied Sciences, Ulm, Germany

**Keywords:** RGBW-LED, Diaphanoscopy, Retinal hazard, Correlated color temperature, Intraocular illumination

## Abstract

Diaphanoscopic illumination has the disadvantage that the intraocular spectrum is red-shifted due to transmission properties of the eyewall. This red-shift should be counteracted as well as the retinal risk should be reduced with adjusting the spectral distribution of the illumination light. Likewise, the illumination spectrum has to be adapted to the eye color of the patient. With the further development of a red, green, blue and white light-emitting diode (RGBW-LED) diaphanoscopy pen, the intensities of each color can be varied. The functionality of the LED pen is tested on ex-vivo porcine eyes. By measuring the transmission of the sclera and choroidea, the photochemical and thermal retinal hazard and the maximum exposure time are determined according to the standard DIN EN ISO 15004-2:2007. With this RGBW-LED pen the intraocular space can be illuminated clearly of up to 1.5 h without potential retinal damage according to DIN EN ISO 15004:2-2007. By adjusting the illumination spectrum the red-shift can be compensated and retinal risk can be reduced. By varying the LED intensities, the correlated color temperature in the eye can also be varied from cold white to warm white appearance as comfortable to the ophthalmologist. Additionally, a simple adjustment of the illumination to the eye color of the patient is possible. Using this RGBW-LED pen, the ophthalmologist can set the desired intraocular color appearance, which he prefers for special applications. He could also adjust the illumination to the eye color as this would reduce retinal hazard.

## Introduction

In diaphanoscopy, the eye is illuminated from the outside through the eyewall. When passing the eyewall, the spectrum of the illumination light experiences a red-shift, so that the ophthalmologist perceives the light inside the eye more reddish, compared to the rather white light of the illumination source. However, diaphanoscopy also has many advantages. It is a patient-friendly and non-invasive procedure to illuminate the eye. Compared to endoillumination, sterility requirements are not as high, because no incisions have to be made to the eye, leading to lower risk of postoperative infections. The light also enters the eye more homogenized and diffuse than with endoillumination due to light scattering in the sclera [1, 2]. In addition, shadows are reduced and unwanted reflexes at the lens are absent. The retinal hazard is even smaller than with endoillumination, since light must be transmitted through the eyewall, and is therefore less intensive as light from endoilluminators that illuminate the retina directly. Due to many advantages and also despite the red-shift in the eye, diaphanoscopic illumination has many applications in the eye, e.g. tumor localization and therapy [3–8], localization of retinal breaks [9, 10], removal of vitreous [11, 12], peeling the internal limiting membrane [13] and diaphanoscopically guided cyclophotocoagulation [14]. The improvement of diaphanoscopic applications keeps going on, e.g. with the integration of light guides in scleral depressors [9–12, 15].

The development of a first red–green–blue-white light-emitting diode (RGBW-LED) diaphanoscope by Busshardt et al. (2021) [16] allowed the eye to be illuminated by different LEDs with different colors, which can vary in brightness, leading to higher contrast images of the tissue and structures of the eye.In our study, the system from Busshardt et al. (2021) [16] is improved by applying LEDs that have higher luminous fluxes. Another improvement that the new system presented in this study should include is an improved heat dissipation and with this a reduced temperature at the tip of the LED pen. Additionally, the evaluation of retinal hazard is improved. Since the LED pen is in direct contact with the eyewall, a risk assessment of the radiation on the retina must be performed. The guidelines in the standard DIN EN ISO 15004-2:2007 and in Sliney et al. (2005) are used for this purpose [17, 18]. For this risk assessment, it is necessary to know how much light is transmitted through the sclera and choroidea in order to determine the actual exposure to the retina. For this purpose, additional transmission measurements of these two layers in combination of ex-vivo porcine eyes are performed. With the transmission properties of sclera and choroidea the potential photochemical and thermal retinal risk is evaluated according to [17, 18]. The risk assessment in [16] was performed using only scleral transmission, neglecting the choroidea, which contains hemoglobin that absorbs much of the dangerous blue light. In addition, the influence of pigmentation is included in our study, which is missing in [16],

The new RGBW-LED pen is tested for functionality on ex-vivo porcine eyes. Additionally, this study also performs further tests on porcine eyes, examining correlated color temperature (CCT) for different combinations and intensities of the various LEDs. CCT has an effect on the visibility and contrast of biological structures, with higher CCT providing a higher image contrast [19, 20]. By adjusting the LED intensities, the physician can vary the CCT of the lighting and also reduce the photochemical and thermal hazard to the patient’s eye. Since the total transmission of the eyewall depends on the degree of pigmentation [21], the retinal hazard, and CCT may also vary for eyes with different degrees of pigmentation. Thus, by adjusting the light intensities, the surgeon can counteract the lower transmission in highly pigmented eyes than in low pigmented eyes, or reduce the increased risk of low pigmented eyes compared to highly pigmented eyes.

Potential application for such am advanced RGBW-LED diaphanoscopic illumination pen include not only the general operating room, but also areas following natural disasters, in which a healthcare infrastructure is lacking, such as after earthquakes, volcanic eruptions, or floods. The treatments there can be performed well with diaphanoscopic examinations, due to the lack of incisions in the eye, there is little requirement for sterility. Compared to conventional lighting systems, the new illumination system provides a low-cost solution for a wide range of applications.

## Materials and methods

### RGBW-LED diaphanoscopy pen

**Fig. 1:**
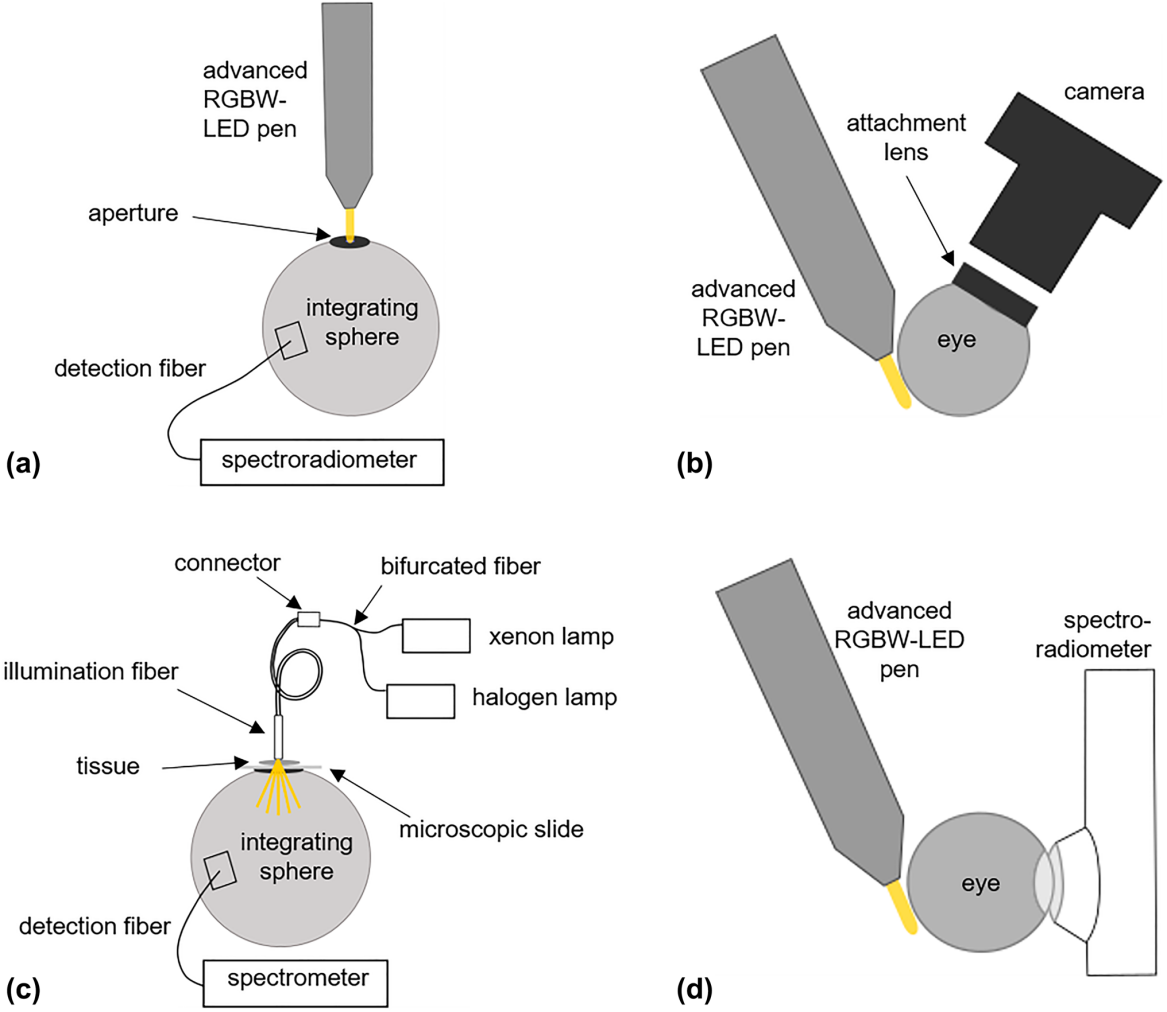
**a** Set-up for determining the emitted irradiance of the advanced RGBW-LED pen. Light was captured by an integrating sphere and detected with a spectroradiometer. **b** Set-up for tests with the advanced RGBW-LED pen on ex-vivo porcine eyes. The RGBW-LED pen illuminated the eye transsclerally. With an attachment lens and a camera the fundus of the eye could be displayed. **c** Set-up for transmission measurement of sclera and choroidea. A section of the sclera and choroidea was illuminated with a fiber and transmitted light was collected with an integrating sphere and detected by a spectrometer. **d** Set-up for determining the CCT the ophthalmologist perceives during surgery. The eye was illuminated transsclerally by the advanced RGBW-LED pen and light in front of the pupil was detected by a spectroradiometer. (Color figure online)

As diaphanoscopic light source an SMD-LED XMLDCL-00-R250-00C5AAA02 LED (Cree, Durham, USA), including a red, green, blue and white LED (RGBW-LED) was used with peak wavelengths of 640 nm, 520 nm, 450 nm and 445 nm (for the blue peak of the white LED). The white LED had a CCT of 6000 K. The dimensions of the LED was 5 mm × 5 mm and it was mounted on a copper board, which was connected to a copper bar to dissipate the heat from the LEDs. For further heat reduction a 33.5 mm long light pipe PLP5-1000 (Bivar, Irvine (CA), USA) was placed close to the LEDs. The tip of this light pipe was applied for transscleral illumination of the fundus and was thus in contact with the sclera. Light pipe, LED board, copper bar and power cables were positioned using a 3D-printed framework. The design of these components are new compared to the existing RGBW-LED pen in [16], resulting in much better heat dissipation and therefore higher maximum LED intensities. The aluminum housing of the pen was 25 mm in diameter. Since too much heat is harmful to the sclera, the temperature was measured at the tip of the light pipe. The thermometer employed was the UT320 (UNI-T, Dongguan City, China). The control unit and the handling of the LED pen was similar to [16]. One improvement is the new minicomputer to control the LED intensities, Pi 4 Model B minicomputer (Raspberry Foundation, Cambridge, Great Britain). The current applied at each LED could be controlled independently in 5% steps with “ + ” and “ − “ buttons. 100% corresponds to a current of 350 mA. The spectral irradiance ${\text{E(}}\uplambda {\text{)}}$ emitted by the RGBW-LED pen was measured with the calibrated spectroradiometer CAS 140D, which was connected to an integrating sphere (ISP 250 UV, Instrument Systems, Munich, Germany), illustrated in Fig. 1a. The aperture of the integrating sphere was 3 mm in diameter, representing the contact surface of the diaphanoscopic pen on the eye.

### Test on ex-vivo porcine eyes

For the test of the RGBW-LED pen, porcine eyes from a local butcher were used. Measurements were performed on the day of enucleation. The eyes were stored in balanced salt solution at 4 °C in the fridge and were divided in two groups, eyes with brown/dark iris and eyes with blue/light iris. As the iris color is correlated to the amount of melanin in the choroid-retinal pigment epithelial cells [22], the eyes were called strong and less pigmented in our study. The transscleral illumination tests on porcine eyes were performed with single LEDs or LEDs in combination. If a LED was turned on the intensity was set to 100% (corresponding to 350 mA LED current). The pen was placed at the posterior pole of the eye and light was transmitted through the eyewall into the interior space. This light was detected and sharp images were generated with a camera (D5600, Nikon, Tokio, Japan), in front of the porcine lens (Fig. 1b). In addition, a biconvex attachment lens was placed on the cornea so that the retina could be brought into focus. The camera exposure time, HDR setting as well as aperture were kept constant when photographing the eye. Only the ISO value of the camera was adjusted to the light conditions of the transscleral illumination.

### Retinal hazard evaluation

With the wavelength dependent irradiance of the LED pen, ${\text{E(}}\uplambda {\text{)}}$ , the photochemical and thermal hazard to the retina could be calculated. According to the international standard DIN EN ISO 15004-2:2007 [17] and Sliney et al. (2005) [18] the photochemical hazard was calculated by weighting the irradiance ${\text{E(}}\uplambda {\text{)}}$ with the photochemical hazard weighting function A(λ), see Eq. (1). E_A−R_ is called the “with A(λ) weighted irradiance on the retina”. The photochemical hazard weighting function is high in the low wavelength region, therefore the sum in Eq. (1) had to be calculated between 305 and 700 nm. For determining the thermal retinal hazard, the irradiance was weighted with the thermal hazard weighting function R(λ), see Eq. (2). E_VIR-R_ is called the “with R(λ) weighted visible and infrared irradiance on the retina”. As the thermal hazard is the highest in the visible and infrared region, the sum in Eq. (2) ranges from 380 to 1400 nm. The standard for ophthalmic instruments EN ISO 15004-2:2007 specifies limit values for these photochemical and thermal weighted irradiances. The limit values for the photochemical hazard is 0.22 mW/cm^2^ and for the thermal hazard 700 mW/cm^2^ [17, 18]. No retinal damage is to be expected below these values. If the photochemical limit of 0.22 mW/cm^2^ was exceeded, the maximum exposition time had to be considered and was calculated as in Eq. (3).

1 $${\text{E}}_{{{\text{A}} - {\text{R}}}} = \mathop \sum \limits_{{305\;{\text{nm}}}}^{{700\;{\text{nm}}}} {\text{E(}}\lambda {)} \cdot {\text{A(}}\lambda {)} \cdot \Delta \lambda$$

2 $${\text{E}}_{{{\text{VIR}} - \;{\text{R}}}} = \mathop \sum \limits_{{380\;{\text{nm}}}}^{{1400\;{\text{nm}}}} {\text{E(}}\lambda {)} \cdot {\text{R(}}\lambda {)} \cdot \Delta \lambda$$

3 $${\text{t}}_{{\text{max }}} = \frac{{10{\text{ J}}/{\text{cm}}}}{{{\text{E}}_{{{\text{A}} - {\text{R}}}} }}$$

Since light is attenuated by the sclera and choroidea before it hits the retina, the photochemical and thermal hazard were also reduced. To determine the actual exposure on the retina, the irradiance ${\text{E(}}\uplambda {\text{)}}$ was weighted by the transmission ${\text{E(}}\uplambda {\text{)}}$ of the sclera and choroidea. Equations (1–3) then were replaced by Eqs. (4–6).

4 $${\text{E}}_{{{\text{A}} - {\text{R,}}\;{\text{Trans}}}} { = }\mathop \sum \limits_{{305\;{\text{nm}}}}^{{700\;{\text{nm}}}} {\text{E(}}\lambda {)} \cdot {\text{T(}}\lambda {)} \cdot {\text{A(}}\lambda {)} \cdot \Delta \lambda$$

5 $${\text{E}}_{{{\text{VIR}} - {\text{R,}}\;{\text{Trans}}}} { = } \mathop \sum \limits_{{380\;{\text{nm}}}}^{{1400\;{\text{nm}}}} {\text{E(}}\lambda {)} \cdot {\text{T(}}\lambda {)} \cdot {\text{A(}}\lambda {)} \cdot \Delta \lambda$$

6 $${\text{t}}_{{\text{max,Trans}}} = \frac{{10{\text{ J}}/{\text{cm}}}}{{{\text{E}}_{{{\text{A}} - {\text{R,Trans}}}} }}$$

The transmission of the sclera and choroidea was determined as illustrated in Fig. 1c. Light from a xenon lamp LQX 1000 SC (Linos, Goettingen, Germany) and a halogen lamp SLS201 L/M (Thorlabs, Newton, USA) were combined with a bifurcation fiber BFY400MS02 (Thorlabs, Newton, USA) and coupled into an ophthalmological illumination fiber TotalView Endoillumination Probe including illuminated scleral depressor 3269.B06 (D.O.R.C., Zuidland, The Netherlands). The fiber tip was in contact with the tissue and light, which was transmitted through the tissue, was captured by the integrating sphere MSP REFLTRANS1 (Mountain Photonics GmbH, Landsberg am Lech, Germany) and detected with a spectrometer AvaSpec-HSC 1024 × 58TEC-EVO (Avantes, Apeldoorn, The Netherlands), which was connected to the integrating sphere via another fiber M114L01 (Thorlabs, Newton, USA).

### Correlated color temperature

To determine the CCT the surgeon perceives during surgery for various settings of intensities for different LED combinations a spectroradiometer BTS256-LED (Gigahertz-Optik GmbH, Türkenfeld, Germany) with an aperture of 10 mm diameter was placed in front of the eye. The inside of the eye was illuminated transsclerally with the RGBW-LED illumination pen with various combinations and intensities of the LEDs. A schematic sketch is illustrated in Fig. 1d). The CCT was provided by the software of the spectroradiometer.

## Results

### RGBW-LED diaphanoscopy pen

**Fig. 2:**
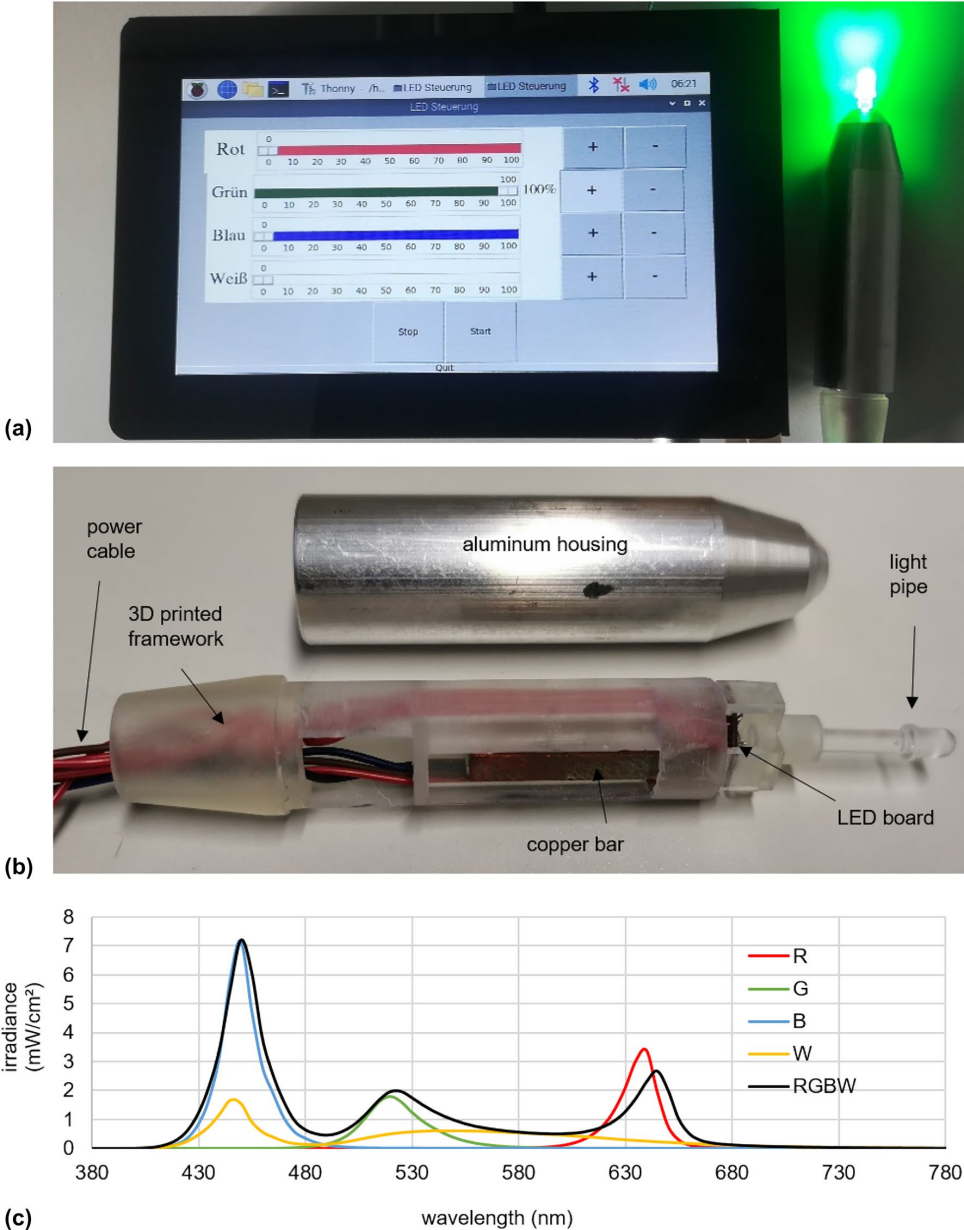
**a** Graphical user interface of the RGBW-LED pen with the green LED set to 100%. **b** Components of the RGBW-LED pen. **c** Spectral irradiance of the red (R), green (G), blue (B) and white (W) LED at 100% and all LEDs combined (RGBW) at 100%. (Color figure online)

The final RGBW-LED pen is displayed in Fig. 2a with the green LED set to 100%. A more detailed presentation of the pen is given in Fig. 2b. A 3D printed framework connects and attaches the power cables, the copper bar, the LED board and the light pipe. The aluminum housing, which envelopes them all, is shown separately. The spectral irradiance for the red, green, blue and white LED and all LEDs together, RGBW, at 100% are given in Fig. 2c. If all 4 LEDs are operated simultaneously at 100%, the temperature at the tip of the light pipe is 30.2 °C.

### Test on ex-vivo porcine eyes

**Fig. 3:**
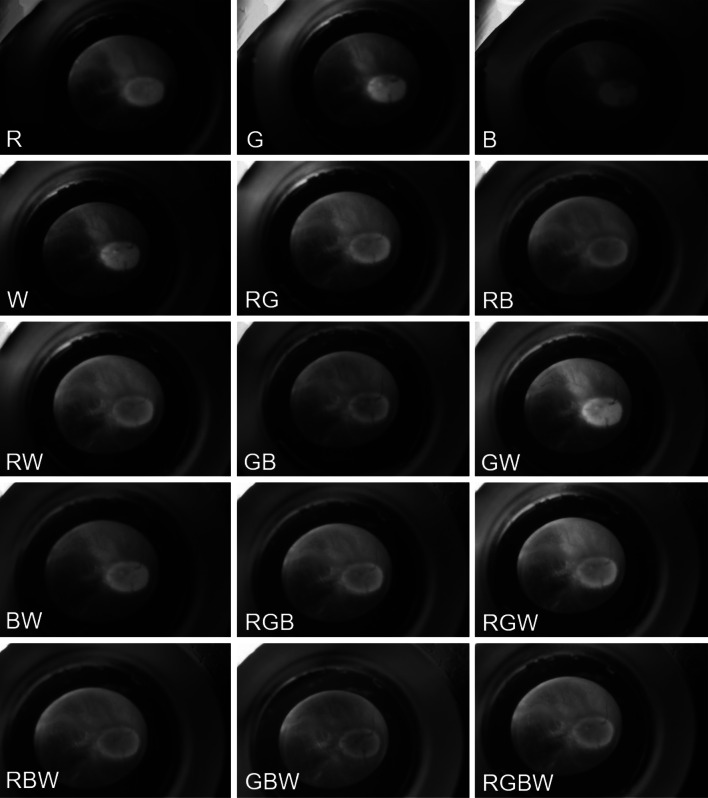
Fundus images of ex-vivo porcine eyes transsclerally illuminated with the RGBW-LED pen for different LED combinations at 100% intensity for each LED. The optic nerve is clearly displayed in the center of each image. Additionally, vessels can be visualized much better for example with green and white (GW) illumination than with red (R) illumination. (Color figure online)

The images of the transsclerally illuminated fundus taken with the camera are presented in Fig. 3. The LEDs are set to 100% intensity and were turned on individually and in combination to illuminate the eye. Depending on the illumination setting, the retina with its blood vessels and optic nerve (brighter oval area on the right side of the retina) are more or less visible. For example, the image with green and white illumination light (GW) makes vessels more visible than with red illumination light (R).

### Retinal hazard evaluation

**Fig. 4:**
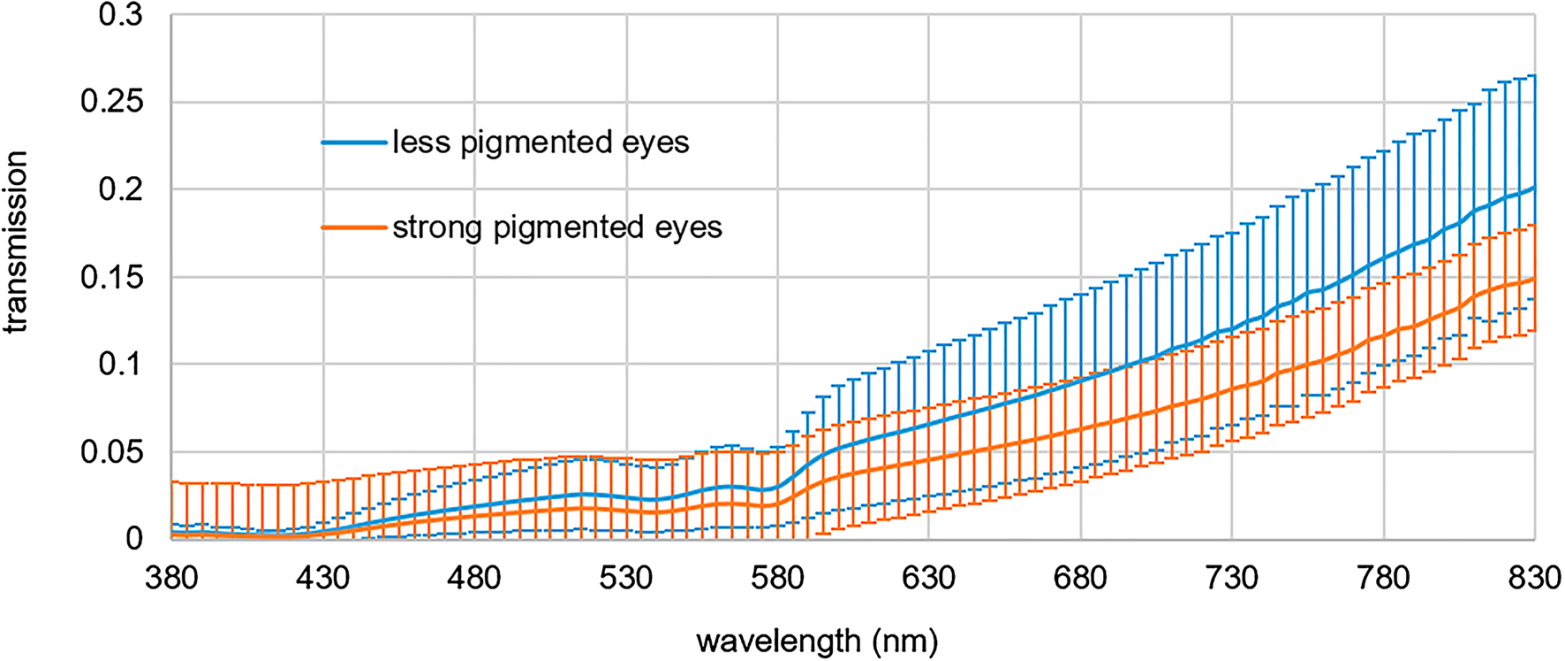
Mean transmission of sclera and choroidea of eyes with less pigmentation (blue curve) and strong pigmentation (orange curve) with standard deviation in the range between 380 and 830 nm. (Color figure online)

The photochemical and thermal hazard and the maximum exposition time of the RGBW-LED pen are calculated according to Eqs. (1–3). These values would apply when the pen is directly on the retina. However, since the transmissions of the sclera and choroidea attenuate the light, the hazards and the maximum exposure time are calculated according to Eqs. (4–6). The mean transmission of sclera and choroidea, which is used in Eq. (4–6), is presented in Fig. 4 with corresponding standard deviation. The blue curve illustrated the transmission of eyes with less pigmentation (blue iris) and the orange curve illustrates the transmission of eyes with strong pigmentation (brown iris). It can be observed that the transmission of less pigmented eyes is higher than the transmission of strong pigmented eyes. The transmission is the smallest in the blue wavelength range and increases with increasing wavelength.

The results of all 6 equations from all LEDs with all possible combinations at 0% and 100% intensity are listed in Table 1. Without considering the absorption properties of the eyewall, the limit value for the photochemical hazard is exceeded for all LEDs and LED combinations except for the red LED. The resulting maximum application times t_max, Trans_ are also listed. The thermal limits are not exceeded. Considering the absorption properties of the eyewall, the limit value for the photochemical retinal hazard is partly exceeded. The limit value for the thermal hazard is never exceed. It is observed that E_A-R, Trans_ of slightly pigmented eyes is about 1.44 times higher than that of strongly pigmented eyes.

**Table 1:** E_A-R_, E_VIR-R_ and t_max_ for different LEDs and combinations at 100% intensity (without absorption of sclera and choroidea, unrealistic) and E_A-R, Trans_, E_VIR-R, Trans_ and t_max, Trans_ for different LEDs and combinations at 100% intensity with considering the absorption of sclera and choroidea for high and low pigmented eyes

	Without absorption			With absorption (low pigmentation)			With absorption (high pigmentation)		
LEDs	E_A-R_ (mW/cm^2^)	t_max_ (min)	E_VIR-R_(mW/cm^2^)	E_A-R, Trans_ (µW/cm^2^)	t_max, Trans_ (h)	E_VIR-R, Trans_ (mW/cm^2^)	E_A-R, Trans_ (µW/cm^2^)	t_max, Trans_ (h)	E_VIR-R, Trans_ (mW/cm^2^)
R	< 1	2134	76	5	537	5	4	777	4
G	3	54	64	73	38	2	50	55	1
B	141	1	158	1482	2	2	1028	3	1
W	42	4	12	416	7	4	287	10	3
RG	3	54	133	75	37	6	52	53	4
RB	136	1	226	1452	2	7	1008	3	5
RW	41	4	193	418	7	9	289	10	6
GB	138	1	214	1503	2	3	1043	3	2
GW	43	4	180	470	6	5	325	8	4
BW	178	1	279	1864	1	6	1293	2	4
RGB	133	1	274	1472	2	8	1023	3	5
RGW	44	4	251	487	6	10	337	8	7
RBW	17	1	345	1869	1	10	1297	2	7
GBW	175	1	331	1891	1	7	1312	2	5
RGBW	168	1	379	1839	2	11	1278	2	8

**Fig. 5:**
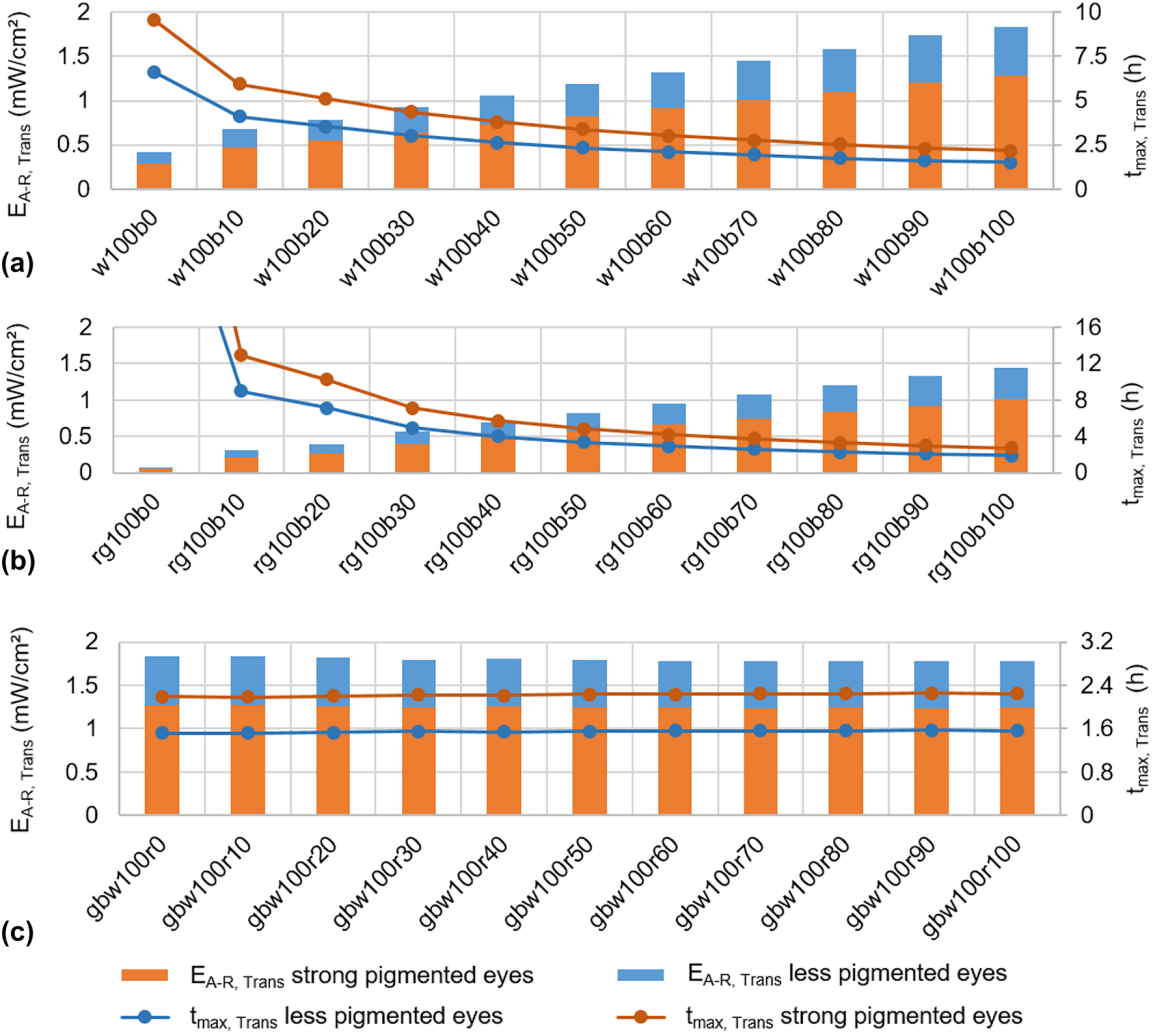
E_A-R, Trans_ (left y-axis) and t_max, Trans_ (right y-axis) for different combinations of the RGBW-LED pen at different intensities. **a** White LED at 100% adding the blue LED in 10% steps. **b** Red and green LED at 100% intensity and adding the blue LED in 10% steps. **c** Green, blue and white LED at 100% intensity and adding the red LED in 10% steps. Blue and orange bars indicate the results for less and strong pigmented eyes. (Color figure online)

Varying LED intensities results in different values for E_A-R, Trans_ and in different allowed application times. The following variations in LED intensities are given by, for example, w100b50. This means the white LED is set to 100% and the blue LED to 50% intensity. The letter indicates the LED color and the digit after the letter indicates the intensity of the LED. If a color does not appear in the letters, the corresponding LED is off. If the white LED is operated at 100% intensity the blue LED can be added in discrete steps to compensate the red-shift. In Fig. 5a, E_A-R, Trans_ for strong pigmented eyes is given in orange and for less pigmented eyes in blue. With increasing blue component, E_A-R, Trans_ increases (left y-axis) and t_max_ decreases (right y-axis). It is also noticed, for example, that when examining low pigmented eyes with 100% intensity of white LED and 50% intensity of blue LED, the hazard is even a little higher than examining high pigmented eyes with 100% white and 100% blue LED. Figure 5b presents the behavior if the RGB-LEDs are set to 100% and the blue LED is reduced by discrete intensity steps. Reducing the intensity of the red LED also reduced the red-shift of light by transmitting the eyewall but would not affect E_A-R, Trans_, see Fig. 5c.

### Correlated color temperature

**Fig. 6:**
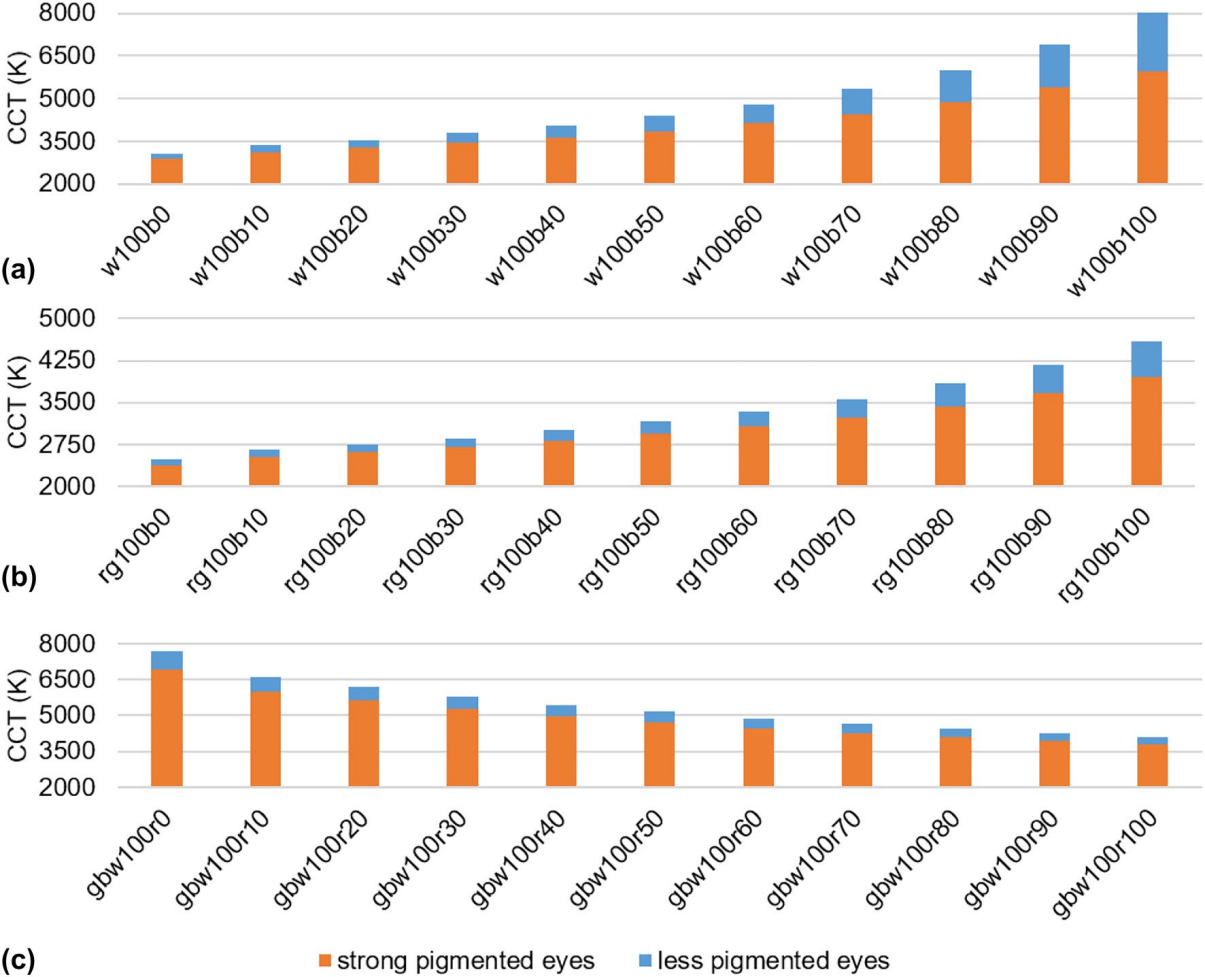
CCT in front of the eye pupil for different combinations of the RGBW-LED pen at different intensities. **a** White LED at 100% adding the blue LED in 10% steps. **b** Red and green LED at 100% intensity and adding the blue LED in 10% steps. **c** Green, blue and white LED at 100% intensity and adding the red LED in 10% steps. Blue and orange bars indicate the results for less and strong pigmented eyes. (Color figure online)

By varying the intensities of the LEDs analogue to Fig. 5 the CCT also changes. Adding the blue LED (100%) to the white LED (100%) increases the CCT from around 3000 K to around 8000 K (Fig. 6a). There are also differences in CCT for different pigmented eyes. For example, in Fig. 6b, it is evident that the CCT at a setting of rg100b60 for less pigmented eyes is almost the same as for rg100b80 for highly pigmented eyes. The intensity of the blue LED can be reduced by 20% and with this the hazard to the retina decreases. If all LEDs are set to 100% and the intensity of the red LED is reduced, the red-shift of the intraocular illumination can be counteracted and the CCT increases as the red content decreases (Fig. 6c).

## Discussion

The application of diaphanoscopic illumination can be further advanced using the improved RGBW-LED diaphanoscopic pen. This pen enables intraocular illumination of the eye with red, green, blue and white light using the RGBW-LED (Fig. 3). These LEDs can be controlled individually, enabling bright and high-contrast illumination of the various tissues and structures of the ex-vivo porcine eye and facilitating the diagnosis of pathological changes. With the blue LED, for example, the fluorescent dye fluorescein can be excited in order to perform fluorescein angiography [23] and the visualization of the vitreous is improved [24]. With green illumination (red-free) the anterior retinal structures are highlighted and with red light illumination choroidal vasculature can be emphasized [25]. Adjusting the color to make the fundus more yellow/orange results in improved staining of the internal limiting membrane with brilliant blue G stain and with adjusting the color to make the fundus appear more red improves the visualization of indocyanine green stain [24]. The nerve fiber layer is best seen under green illumination, the retinal vessels are best visualized under yellow illumination, the granular texture of the retinal pigment epithelium under orange/red light and the yellow macular pigment are best seen under blue illumination [26, 27]. Ophthalmologists have different color perception and different preference in color temperature and color appearance in the patient's eye. With the adjustable RGBW-LED pen, the spectrum can be tuned as it appears the best to the ophthalmologist. In addition, the adjustable RGBW-LED pen can compensate for the red-shift in intraocular illumination [28, 29] that occurs due to the transmission properties of sclera and choroidea (Fig. 4). With increasing the intensity of the blue or white LED or with dimming the red LED this is feasible. In Figs. 3 and 6 it becomes apparent that CCT increases with higher blue or white content or with lower red content, resulting in whiter illumination with less red-shift. With increasing LED intensities, especially the blue and white LED intensities, the hazard to the retina also increases (Fig. 5). Despite to the low transmission of the sclera and choroidea, the limit for the photochemical hazard specified in DIN EN ISO 15004–2:2007 [17] is partly exceeded. For this, the corresponding exposition time should be respected and should not be exceeded. As the average ophthalmic surgery lasts around 110 min [30] these times would not be exceeded anyway. The limit value for the thermal hazard is not exceeded.

This study is an extension to the study by Busshardt et al. (2021) [16], which considered the hazard evaluation of the diaphanoscopy pen at its tip and under consideration of the scleral transmission only. However, since hemoglobin has a very high absorption in the blue wavelength range [31], this has a major impact on retinal hazard. Therefore, in this study, the transmission properties of sclera and choroidea were investigated combined and included in the hazard calculation. Transmission of sclera and choroidea together was not published before, but is important for calculating retinal load during transscleral illumination. The temperature on the tip of the diaphanoscopy pen is improved compared to the study of Busshardt et al. (2021) [16] and is reduced to around 30 °C. This is due to the improved heat dissipation with the help of the copper bar, which is in contact with the LED board, and with the long 33.5 mm light pipe in front of the LED, compared to the self-made 20 mm long light pipe in [16] where 37 °C was measured at the tip. An extension of our study is the consideration of the pigmentation of the eye. For eyes with different degrees of pigmentation, a different setting of the RGBW-LED pen can be chosen to obtain an equally good illumination of the eye fundus. Another improvement is the higher luminous flux of the LEDs. In comparison to Busshardt et al. (2021) where the RGBW-LED pen reached 15 lm for maximum possible intensities measured with an aperture of 0.68 mm diameter (with the set-up presented in Fig. 1a), in our study the advanced pen reaches 48 lm for all LEDs set to 100% intensity with the same aperture. Thus, the eye can be illuminated more brightly with the pen developed in our study compared to the pen of Busshardt et al. (2021). Likewise, several color variants are pictorially represented than only red, green, blue and white. The cold white LED applied in our study has a color temperature of 6000 K, whereas the warm white LED in [16] has 3700–4300 K. The cold white LED applied in our new system improves intraocular vision by compensating for the red-shift with the higher blue intensity, which allows better visibility of intraocular structures. A further extension of [16] is the investigation of the influence of LED intensity and spectral distribution on intraocular CCT. In order to visualize structures more effectively, a lower red component would be useful. Comparing the CCT of highly and lightly pigmented eyes, it can be noticed that by illuminating lightly pigmented eyes at a setting of 80% intensity of the red LED and 100% of the green, blue, and white LEDs (gbw100r80) provides about the same CCT as 100% intensity of the red LED (gbw100r100) in highly pigmented eyes (Fig. 6c). Similarly, reducing the amount of blue can cause the same CCT for low-pigmented eyes as for highly pigmented eyes with full blue LED power. For example, reducing the blue component by 20% (rg100b80) for lightly pigmented eyes will produce approximately the same CCT as for highly pigmented eyes without blue light reduction (rg100b100) (Fig. 6b). By adjusting the LED intensities, highly pigmented and lightly pigmented eyes can thus be equally well illuminated. The adjustment of intensities, in the comparison of highly and poorly pigmented eyes, also plays a major role in retinal risk, as the hazard can thus be reduced for less pigmented eyes. If the intensity of the blue LED is reduced by about 20% (rg100b80) when illuminating less pigmented eyes, the photochemical hazard decreases in comparison to rg100b100 and approaches the value that strongly pigmented eyes exhibit at 100% blue LED intensity (Fig. 5b). The intensity of the blue LED can be lowered by 20% for blue eyes, for example, thus reducing the risk to the retina while maintaining the same CCT as for brown eyes.

In this respect, the adjustable intensity of the red, green, blue and white LEDs is not only useful for different applications and visualization of structures in the eye, but also in the adaptation to the patients’ eye color. Retinal risk can be reduced by adjusting the LED intensities according to the pigmentation of the eye. As the human sclera is somewhat thinner than that of porcine eyes [32] and the melanin content in the human eyewall is lower than in porcine eyes [22, 33] the transmission would decrease and the photochemical and thermal retinal hazard would increase. The allowed maximum exposure times for transscleral illumination are relatively high and in the range of a few to several hours (Table 1), however, the surgeon should still vary the position of the LED pen to be on the safe side, so that the same area of the retina is not always directly illuminated. Furthermore, a timer could be integrated into the next prototype, which indicates when the limit value from DIN EN ISO 15004-2:2007 [17] is reached and alerts the physician by beeping.

## Conclusion

With the RGBW-LED diaphanoscopy pen, the ophthalmologist is able to set the desired intraocular color appearance and intensity which he personally prefers for different applications. With varying LED intensities the ophthalmologist is able to compensate the red-shift of the intraocular light spectrum. The intraocular space is illuminated clearly and there is no potential photochemical or thermal retinal risk according to DIN EN ISO 15004:2–2007, considering the maximum application time. When all LEDs are set to 100% intensity, the eye can be illuminated for up to 1.5 h. With this device, the ophthalmologist can adjust the LED intensities to the patient´s eye color. Lowering LED intensities would reduce retinal risk for patients with blue eyes compared to brown eyes. Since this device is very inexpensive compared to conventional ophthalmic devices, and has fewer sterility requirements than endoillumination, this device can be well applied in developing countries.
